# Regulatory Intensity on Private Forestland and its Relationship with State Characteristics in the United States

**DOI:** 10.1007/s00267-024-01974-6

**Published:** 2024-05-06

**Authors:** Kamana Poudel, Mindy S. Crandall, Erin Clover Kelly

**Affiliations:** 1 Department of Forest Engineering, Resources and Management, Oregon State University, Corvallis, OR 97331 USA; 2 Department of Forestry, Fire, and Rangeland Management, Cal Poly Humboldt, Arcata, CA 95521 USA

**Keywords:** Regulations, Corporate forestland, Private forests, Forest policy, Forest management

## Abstract

Though the federal government impacts private forest management across the United States through legislation such as the Clean Water Act, state-level regulations applied to private forest landowners vary remarkably. Despite this diversity of policies, little is known about how variations in regulatory intensity (defined here as number of forestry regulations) correlate with state-level political and socioeconomic characteristics. In this study, we use a quantitative approach to explore the intensity of regulation on forest practices impacting private landowners across all 50 states. We quantified intensity by tabulating the number of regulated forest practices, then used a quasi-Poisson regression to estimate the relationship between regulatory intensity and state-level characteristics, including forestland ownership types, the economic importance of the forest industry, and measures of state environmentalism. Results indicated a positive association between regulatory intensity and the percent of private corporate land, environmental voting records of elected officials, and direct democracy. Foresters and landowners may learn from these relationships, consider how to influence different policies, and build or achieve greater levels of public trust. This study starts to help us explain *why* state-level forestry policies differ, not just how they differ.

## Introduction

Although globally, most forestland is in public ownership, some countries or regions have substantial amounts under private ownership, including the United States (Oswalt et al. 2019) and Europe (Schmithüsen and Hirsch 2010). Around 58% of the forestland of the United States is privately owned by individuals, families, partnerships, corporations, non-government organizations, and other groups (including Native American Tribes) (Oswalt et al. 2019). These privately owned forests provide a wide array of public goods and services, ranging from clean water and timber to wildlife habitats and recreational opportunities, as well as the bulk of the commercial timber supply (Stein et al. 2009), and the provision of goods and services from private forestland depends on sustainable management of the private forestland (Butler et al. 2016).

Despite strong private property rights in the United States that provide landowners broad latitude in the activities they can conduct, many states within the United States have a long-standing history of regulating management activities on private forestland. The authority of states to constrain private management actions to protect public goods and services was established in a Washington State court decision in 1947 (Ellefson and Cubbage 1980). The Federal Water Pollution Control Act (Clean Water Act, CWA) of 1972 requires states to protect water quality and mitigate water quality issues arising from forest practices; however, states have flexibility to choose policy mechanisms ranging from regulatory to voluntary to accomplish this. The tension between private rights and the state’s responsibility to protect public goods has been reflected both in the types of policies implemented by each state and in the language used to describe and justify their implementation (Kelly and Crandall 2022; Goldstein et al. 2023a). Following the introduction of Best Management Practices (BMPs) in the 1977 amendment of the CWA, many states have implemented a variety of predominately voluntary policies, including voluntary BMPs, technical assistance and education programs, and tax incentives.

While voluntary landowner participation and incentives are important, they may be insufficient or perceived as insufficient to protect the myriad of ecosystem values forests provide; some states instead rely on command-and-control, top–down approaches termed “regulatory” (Böcher 2012). The earliest regulations on private forestland were frequently related to the reforestation of cut over forestlands, typically through requiring the maintenance of seed trees; seed tree requirements were enacted in 1941 in Oregon and in 1950 in Virginia (Code of Virginia 1950§10-74.1 to -83.01; Cubbage 1991; Ellefson et al. 1997a). Since then, some states have passed more comprehensive State Forest Practice Acts (FPAs), particularly during the rise of environmental policies at both the federal and state levels and as a response to the CWA. Significant FPAs include Oregon’s Forest Practices Act (originally enacted in 1971) and California’s Z’Berg-Nejedly Forest Practice Act of 1973. Today, emerging issues include regulations or provisions related to aesthetic, carbon, and wildlife habitat concerns (Ellefson et al. 2007; Thompson and Hansen 2012; Tian et al. 2015).

Given the wide latitude of states to utilize different policy tools to achieve sustainable forest management and the provision of public goods, several lines of research have sought to understand the diversity of policies impacting private forestland management across the United States. These key studies, many initially led by Ellefson et al. (1997a, 2007), focused on summarizing the state-level role in regulating forest practices, including the agencies and programs involved and the investment levels of state-administered programs. To categorize the various roles that states have and the policy tools employed, previous literature has generally articulated three dominant approaches to private forestry policies: regulatory/mandatory, quasi-regulatory, and non-regulatory/voluntary systems (Ellefson et al. 1997b; Cristan et al. 2018; National Association of State Foresters 2019). While regulatory and non-regulatory are two ends of a spectrum, the quasi-regulatory approach encourages a landowner to implement BMPs where programs are voluntary; however, if an environmental violation occurs, the landowner may be subject to regulations and penalties. This approach has also been called contingent or conditional (Ellefson et al. 1995).

Other past research on private forestland policies has focused on the perceived effectiveness of state-level BMP programs (Henly et al. 1988; Rose and Coate 2000; Cristan et al. 2016; Kooistra et al. 2018; Kreye et al. 2019) or on the perceptions, attitudes, motivations, and behaviors of forest landowners toward policy programs (Creighton and Baumgartner 2005; Janota and Broussard 2008; Quartuch and Beckley 2014; Kooistra et al. 2018; Kreye et al. 2019; Goldstein et al. 2023b). However, little is understood about the influence of context on the private forestry policy tools used by states, in particular the relationship between state-level policy approaches and political, social, and ecological characteristics of the state. In addition, most of the previous literature focuses primarily on policies related to water quality; while the CWA provided much of the original motivation for state-level policy development, many other forest public goods or management actions may be regulated today.

Outside of the forestry realm, previous literature has argued that political institutions, economic conditions, and environmental characteristics can lead to differences in environmental activism and thus differences in environmental regulations across states (Congleton 1992; Bromley-Trujillo 2012). Studies have shown that states with strong environmental interest groups, presence of large manufacturing, and Democratic leaders, demonstrate support for pro-environmental policies (Bromley-Trujillo 2012). Researchers from outside the United States have also explored how the complex, dynamic interplay of political and economic forces influences property rights (Birben 2019) and have highlighted a need for more research on how property rights play a role in forestry (Solberg and Rykowski 2000; Glück 2002; Irimie and Essmann 2009). Thus, it may be important to consider the prevailing political, economic, and social contexts of different jurisdictions (Nichiforel et al. 2018), and how these variables influence regulations (Irimie and Essmann 2009).

This study builds on previous work (Table 1) delineating and describing the extent of forest policies impacting private landowners. We add to the existing literature on the impacts of policies on private forest landowners in the United States by documenting a second dimension of impact: the intensity (i.e., number) of regulations at the state level. In addition, we consider the relationship of existing policies to social, economic, and political characteristics through an econometric model comparing the state-level intensity of regulations to state-specific characteristics. The research questions that we investigated were:

A. What is the regulatory intensity impacting private forest landowners in each state?
B. Is there a relationship between state socioeconomic or political characteristics and the regulatory intensity (i.e., number) of private forest practices?

**Table 1:** Evolution of literature on regulations impacting private landowners and their relevant findings

Author and date	Relevant findings
Henly et al. (1988)	Forest practice laws place a considerable administrative burden on states and impose significant compliance costs on landowners and timber operators; however, these regulations have resulted in improvements in forest resource conditions and have led to increased levels of reforestation.
Congleton (1992)	Political institutions, rather than resource endowments, are the primary drivers of environmental regulation policies.
Ellefson et al. (1995)	Study that clarified the regulatory to non-regulatory systems in the United States and defined the “contingent” systems as those where landowners are subject to agency-initiated enforcement action when they fail to voluntarily comply with forest practice standards.
Rose and Coate (2000)	Forest reforestation rules in Oregon are among the strictest in the United States and landowners’ compliance with this regulation can be a lesson to other states.
Ellefson et al. (2004)	Regulatory programs cover a wide range of forest practices and can originate with a single policy or a combination of multiple policies and programs.
Ellefson et al. (2007)	Regulatory programs vary among states in regulating private forestry.
Janota and Broussard (2008)	Landowners view their forest as a long-term financial investment. Of family forest landowners, those that own areas of riparian forest and absentee forest owners tend to be more supportive of various policy tools compared to other landowners.
Bromley-Trujillo (2012)	Wealthy states with strong environmental interest groups, large manufacturing presence, and democratic-controlled governments are more likely to support robust environmental policy than other states.
Quartuch and Beckley (2014)	Forest landowners in both New Brunswick and Maine are fairly comfortable with regulations and agree that a combination of incentives and regulations are useful.
Cristan et al. (2016)	Forestry BMPs are effective in protecting water quality and achieving water quality goals established under the Clean Water Act (CWA) when applied correctly.
Cristan et al. (2018)	Updates the categorization of states into regulatory, quasi-regulatory, and voluntary categories based on BMP implementation to protect water quality and mitigate water quality issues arising from forest practices.
Kooistra et al. (2018)	Riparian buffer regulations led to negative socioeconomic outcomes, and neutral or slightly positive ecological outcomes.
Kelly and Crandall (2022)	State forest policies were extended from the previous framework of regulatory, quasi-regulatory, and voluntary to introduce four distinct typologies based on the extent of state intervention.
Goldstein et al. (2023b)	Landowners in California have nuanced views toward the state’s strict regulatory policies, with general acceptance of the policies but significant concerns about financial burdens and regulatory uncertainty.

In this study, we focus solely on regulatory requirements impacting private landowners, and exclude quasi-regulatory (conditional, contingent) and non-regulatory (voluntary) policies. We take this narrow definition in order to highlight policies that clearly restrict forest practices or require actions of landowners in all circumstances; we decided not to count policies that landowners can opt into or avoid. Although somewhat limiting, this definition provides the clearest, most consistent, and most conservative insight into the regulatory burden facing all private forest landowners.

Understanding the regulatory intensity facing private forest landowners is important for several reasons. First, the level of intensity indicates differing operating environments for landowners, which has implications for economic and environmental outcomes (and public good provision) at state and national levels, with methods that can be compared across jurisdictions (Nichiforel et al. 2018). By documenting the level of regulatory intensity by state, we delineate the minimum level of current regulations facing landowners and provide a baseline to measure other definitions or future operating environments against. In addition, understanding the relationship between regulatory intensity and state characteristics provides insights into which socioeconomic and political factors may be influencing the development of regulations. Understanding this relationship may clarify tradeoffs to policy makers when contemplating the appropriate role of the state in balancing private landowner rights with state-level environmental outcomes. Finally, there is a lack of comparative research on multiple regions and countries regarding the link between property rights and regulations (Nichiforel et al. 2018; Birben 2019) and different factors affecting these rights and regulations. These factors and their interconnections with regulatory intensity could serve as a basis for more geographic comparisons and could also provide a model for assessing other private landowner regulations (e.g., agricultural practices).

## Methods

### Data Collection and Variables Used

We analyzed documents from all 50 states to understand regulatory intensity concerning practices on private forestland, similar to the approach taken by Ellefson et al. (2007) and Kelly and Crandall (2022). The list of forest practices categories was based directly on previous studies (Ellefson et al. 2007; National Association of State Foresters 2019). To capture the evolving nature of forest policies and provide preliminary information that may be essential in understanding future changes, we incorporated two additional categories representing potentially emerging issues in forest management: aesthetics and carbon. This resulted in a final list of 12 potential forest practices of interest (Table 2).

**Table 2:** Forest practices, specific guiding questions, and examples as found in documents

Forest practice	Specific guiding question	Examples as found in documents
Water quality	Are there standards for streamside management zones or riparian areas?	For class 1 and 2 streams, the SMZ boundary is at 15.24 m (50′) on slope 35% or less and 30.48 m (100′) on slope >35%. (*Montana Streamside Management Zone Law and Rules, 2006*)
Reforestation	Are there regulations on regeneration standards?	There should be at least 450 trees/acre well distributed on the harvest area with acceptable growing stock trees that are at least 3 feet in height for softwood trees and 5 feet in height for hardwood trees to qualify for overstory removal. (*Maine Chapter 20, Forest Regeneration & Clearcutting Standards*)
Timber harvesting	Is there a maximum clearcut size or slope consideration for harvesting?	Washington limits clearcuts to 120 acres. (*Washington Forest Practices Act*)
Wildlife and endangered species	Are there restrictions on forest management to protect wildlife, or rare, threatened, or endangered species of plants and animals?	Operators must leave at least two standing live trees or snag per acre of harvest (30 feet tall and 11 inch in diameter) to provide important nesting sites and habitat for birds, bats, squirrels, and many other animals. (*Forest Practice Act, Oregon*)
Prescribed burning	Are there regulations regarding burning permits (personnel or permit)?	Prescribed burn practitioners are required to notify at least 24 h before performing a prescribed burning in Kentucky. (*State and Private Forestry Fact Sheet Kentucky 2022*)
Management and planning	Are there regulations requiring preparation of forest management/timber harvest plans?	A detailed plan of operation must be submitted on a form provided by the division at the area of the division with jurisdiction over the area in which operation will occur. (*Alaska Admin Code § 95.220*)
Notification and permit	Are landowners required to notify the state or receive a permit prior to forest harvesting or operation activities?	The timber owner or landowner must notify the department before commencing a forest practice or a conversion of forest. (*Forest Practice Act, Idaho*)
Infrastructure	Are there standards specific to forestry regarding road construction, maintenance, upgrades, or closures, culvert sizing and upgrades, bridges, and stream crossings?	It is required to design bridges and culverts to accommodate at least the 25-year re-occurrence storm event, specification on p15 of BMP-Massachusetts. (*Best Management Practices, Massachusetts*)
Aesthetic	Are there regulations related to recreation, or other aesthetic or cultural consideration?	Some highways are designated as scenic highway (visually sensitive corridors) to maintain roadside trees for the enjoyment of the motoring public while traveling though forestland. In these highways, it is required to leave at least 50 healthy trees of at least 11 inches DBH, or that measures at least 40 square feet in basal area. (*Forest Practice Act, Oregon*)
Carbon	Does the state regulate carbon in forestry activities?	The harvesting of commercial tree species should maintain the capacity of forest resources including above ground and below ground biomass and soil to sequester carbon dioxide emissions sufficient to meet or exceed the states greenhouse gas reduction requirements for the forestry sector. (Z’berg-Nejedly *California Forest Practices Act*)
Forest health	Are there regulations limiting forest management for protection from insects, and disease spread prevention?	^a^N/A
Timber stand improvement	Are there regulations on timber stand improvement actions, such as commercial pruning or thinning?	^a^N/A

^a^Specific regulatory requirements related to forest health and timber stand improvement were not found in any state

We first accessed forestry BMPs of each state through agency websites to help determine if the forest practice of interest was regulatory or voluntary. We also searched for relevant forest practice acts and regulations by querying official websites and documents. These included forestry or other agency rules and regulations as well as online documentation of state statutes. Some states had information centralized at one location (e.g., Department of Forestry) and some had information at multiple agency websites (e.g., Department of Agriculture, Department of Conservation and Recreation, university extension programs). For searching websites and documents, we used forestry keywords and keywords related to each practice of interest such as “streamside management zone”, “riparian area”, “buffer zone”, “regeneration”, “reforestation”, “harvest”, clearcut”, “slope”, “wildlife”, “endangered species”, “prescribed burning”, “permit”, “burn manager”, “personnel”, “notification”, “management and planning”, “harvest plan”, “plan of operation”, “notification”, “operating activities”, “infrastructure”, “roads”, “culverts”, “construction”, “aesthetic”, “carbon”, and “forest health”, then read the rules and regulations for context. We have referred to the most recent BMPs, FPAs, and latest updates from relevant agency websites; these policies were created in different years across the states. Based on this information, we used a binary yes/no approach to identify, for each state, the presence of a state-level regulation related to the forest practices of interest on private forestland for each of the 12 major forest practices (Supplementary Table 1). We classified a *mandatory* regulation that limited the potential management actions of private landowners as a 1, and regulations that only applied in particular circumstances or under certain conditions (quasi-regulatory) or voluntary/non-regulatory measures as a 0. For example, Maine only requires a management plan for landowners enrolled in the Tree Growth Tax Program; since this represents a program a landowner must opt into, this was not counted as a regulation.

In addition, we chose in some cases to focus on one clearly identifiable aspect of a forest practice, although the practice of interest may involve many potential activities. For example, timber harvesting or prescribed burning have multiple facets that may be regulated (types of equipment used, need for types of personnel, and permits or notifications). While focusing on one aspect is a limitation in some cases, it allowed us to clearly define a 0/1 variable for each type of practice for all states.

Our measure of regulatory intensity was the cumulative sum of the 0/1 tally for each of 12 potential regulated forest practices (discussed above and in Supplementary Table 1) for each state. While we used narrow criteria for each forest practice to understand the existence of regulations on private forestland, this definition provides a quantitative snapshot of regulatory intensity across the country. Table 2 details the major forest practice categories used in the study, specific questions we used to guide our designation of the existence of forest practices, and examples of regulatory language found in the documents.

### State Characteristics Influencing Regulatory Environment

The five variables we used to capture the geographic, economic, and political context of each state, along with their specific definitions and sources, are in Table 3. Due to the small size of our sample (50 states), best modeling practices limited us to five independent variables for a regression (Yong and Pearce 2013). The variation inflation factor for the predicted variables was below 2, suggesting no problematic collinearity. The predicted relationship and rationale behind the inclusion of each independent variable is detailed below.

**Table 3:** Independent variables, their definitions, and sources

Independent variable	Data year	Definition	Source
Private forestland	2017	Private forestland as percent of state forestland	Oswalt et al. (2019)
Corporate forestland	2017	Corporate forestland as percent of private forestland	Oswalt et al. (2019)
Economic importance	2016	GDP from forest products as a percent of total economy of a state	Pelkki and Sherman (2020)
Direct democracy	^a^	State allows for citizen-initiated statutes	Ballotpedia website
Political climate	2016	Average score of environmental voting records of congressional representatives for each state	League of Conservation Voters website

^a^The last state that adopted initiated statutes was in 1968 and the number has not changed since

#### Private Forestland

Landowner objectives for forest management vary greatly between private lands and public lands. The practice of forestry and the public expectations or perceptions of landowner behaviors is likely to be quite different in states dominated by one ownership or another; thus, the relative dominance of public or private landownership might influence the development of and support for regulatory policies on private forestlands within a state. States with greater amounts of private forestland may prioritize protection of private property rights as opposed to public trust resources, particularly in areas with strong private landowner advocacy groups. Prioritizing private rights may, in part, ensure a sustainable timber supply by creating a predictable operating environment, especially where private non-corporate landowners dominate.

Conversely, the role of the public in setting and influencing forest policy is much more visible in states with a high proportion of public lands. Since the passage of key environmental laws impacting federal lands (e.g., National Environmental Policy Act of 1970 and Federal Land Policy and Management Act and National Forest Management Act of 1976), scrutiny of federal land management actions and challenges to federal actions in court have increased (Keele and Malmsheimer 2018; Fleischman et al. 2020). This increasing role of private citizens in influencing management actions on federal lands may lead to greater social acceptability and implementation of regulations on private lands in states with a high proportion of federal lands, if residents of such states feel more entitled to a role for citizen engagement and direction of management activities on forests.

Given these considerations, we hypothesized that higher proportions of private forestland would be correlated with a lower number of regulations across forest practices. We calculated the proportion of private land out of all forestlands in 2017 for each state using data from Oswalt et al. (2019).

#### Private Corporate Forestland

The *type* of private land varies widely from state to state. Private non-corporate landowners comprise the bulk of private landowners in many parts of the Midwest, Northeast, and South. Previous research has connected a discourse focused on the inherent land ethic of private non-corporate landowners to the use of voluntary measures as primary forest policy tools (Kelly and Crandall 2022). Private non-corporate landowners are also likely to be direct constituents of policy decision-makers (e.g., elected officials) and reside in or near the land they manage and may directly influence policymaking through that channel.

In contrast, the role of large companies or non-local interests in perpetuating cut-and-run practices in the early part of the twentieth century may loom large in public perceptions (Robbins 1985). In addition, while we are not able to easily compare the use of more controversial management practices by landowner type with certainty, several public debates over the role of harvest activities such as clearcutting have occurred in states with highly visible, large-scale corporate ownerships. Thus, there may be pressure in states with a high proportion of large industrial or corporate forestland to “rein in” corporate behavior through the use of regulations, regardless of the presence of BMPs or enrollment in certification programs (Panwar et al. 2006).

Both the belief in a land ethic for private non-corporate landowners and a mistrust of private corporate landowners may lead to higher number of regulations on private forest management in states with larger shares of private corporate ownership. We used private corporate forestland as the proportion of all private forestland to capture this hypothesis using data from Oswalt et al. (2019).

#### Economic Importance

The economic importance of the forest products industry in a given state’s economy varies widely, from a high of 5.16% of gross state product in Maine to a low of 0.24% in Hawaii (Pelkki and Sherman 2020). States with a high reliance, or long history of economic reliance, on the forest products industry may have incentives to limit regulation on private lands. This pressure may arise from a desire to preserve the profit margins of essential economic actors and to maintain dominance in certain sectors (e.g., plywood in Oregon) by limiting the regulatory burden on forest management, regardless of the dominant type of landowner (Gale et al. 2012). In this case, high economic reliance on the industry may be correlated to fewer regulations on forest activities.

We used the share of gross domestic product (GDP) from forest product industry, calculated as a proportion of the industry value added to the total economy of a state, to understand the influence of economic importance from forest products on regulation of private forest practices. We hypothesize that the higher the proportion of forest product industry GDP, the lower the regulation intensity on forest practices. Typically, a limiting factor in including this type of data would be the need to conduct economic contribution studies for all states or the need to have consistent methodologies and data years for independent studies, aspects frequently precluded by cost or time. Pelkki and Sherman (2020) assessed the economic contribution of the forest sector for all 50 states using 2016 information; we used their reported information for forest products sector value added and state GDP.

This variable is by necessity a conservative estimate of the value of forests to state economies; it considers only the value of market uses of wood forest products, not GDP value accruing from forest-based tourism, recreation, or carbon markets. While many states may have significant portions of their economy linked to forest-based tourism, estimates of the amount of that economic activity directly tied to forests (as opposed to beaches, or other reasons for visitation) are difficult to obtain. In addition, forest products industry lobbying and advocacy groups are the most likely to work to influence political processes related to regulations on forest management activities. For these reasons we limited our analysis to GDP from the forest product industry.

#### Direct Democracy

Direct participation of citizens in political decision-making can play an important role in creating, amending, or repealing state laws or constitutional provisions; this type of direct democracy varies state to state (Black et al. 2016). In some states, the process for amending state statute includes the ability for citizen-initiated ballot measures to be put forward, voted on by the general public, and become law (Ballotpedia 2021). As the public has become increasingly aware of the non-market goods and services produced by forests, they may also become increasingly engaged in advocating for limitations on forest management activities. For example, the ballot initiative process in California and Oregon has been used by the public to bring forth concerns over forestry practices and has led to forest management reforms and development of new regulations on private forestlands directly through public engagement (Shindler and Cramer 1999; Poudyal et al. 2015).

Data indicating whether states allow for citizen-initiated statutes were retrieved from the Ballotpedia website (Ballotpedia 2021). These states represent citizen direct democracy; we assign binary variables of 1 for states that allow citizen-initiated statutes (21 out of 50) and 0 otherwise. We hypothesize that direct democracy may be associated with higher regulations on private forestland, as citizens concerned with environmental protection can bypass legislative, agency, or state-level board protocols and enshrine public sentiment in law.

#### Political Climate

Many states have clear patterns in electing legislators from the Democratic or Republican party, people who are likely to vote along established party lines with respect to environmental issues. Similarly, states with “pro-environmental” worldviews have been found to have a higher level of concern for the environmental impacts of forest practices in private lands, which may translate into increased intensity of regulations impacting private forestland (Poudyal et al. 2015).

To understand how political climate was related to regulations in forest practices, we used the average score of environmental voting records of the members of Congress for each state. We extracted data from the League of Conservation Voters (LCV 2021) for year 2016 (second session of the 114th Congress). We used 2016 data to be consistent with the GDP variable. LCV scores legislators based on their support of environmental policies related to issues such as clean air and water, energy, climate change, environmental justice, public health, and other environmental programs, on a scale of 0–100 (LCV 2021). The scorecard represents the consensus of experts from more than 20 organizations representing environmental, environmental justice, public lands, and wildlife conservation interests. The vote is a subjective measure of legislators’ support for environmental protection. We focused on elected officials in the House of Representatives, as there is more variation within each state of party representation (and thus voting); we averaged together the scores for all Representatives within a state. We hypothesize that the higher the LCV voting score, the more likely a state has a higher number of regulations.

### Statistical Analysis

Regression analysis was used to understand the relationship between the current level of regulations in private forestland management and state characteristics. The dependent variable, regulatory intensity, was a count variable ranging from 0 to 9 with highly left skewed data. Count distribution such as Poisson distribution can be used to deal with such data (Green 2021) as correlation will not be robust in the case of a categorical dependent variable. However, using Poisson distribution, all the response variables showed overdispersion; thus, we applied the quasi-Poisson distribution and logit link function to test the influence of the proportion of private forestland, proportion of corporate forestland, share of GDP from the forest industry, existence of direct democracy, and environmental voting record on the aggregated level of regulations impacting private forestland management (Eq. 1). We used R studio version 2022.12.0 (R Core Team 2022). We used dplyr (Wickham et al. 2019) package to clean and process the data, and skimr (Waring et al. 2022) to summarize the data. The regression equation is:

1 $$\log ({\rm{E}}({{\rm{regulatory}}\; {\rm{intensity}}}))={\rm{\beta}}0+{\rm{\beta}}1({{\rm{Private}}\; {\rm{forestland}}})+{\rm{\beta}}2({{\rm{Corporate}}\; {\rm{forestland}}})+{\rm{\beta}}3({{\rm{Economic}}\; {\rm{importance}}})+{\rm{\beta}}4({{\rm{Direct}}\; {\rm{democracy}}})+{\rm{\beta}}5({{\rm{Political}}\; {\rm{climate}}})$$

where *β*0 is the intercept and *β*1 to *β*5 are the regression coefficients for the respective independent variables.

## Results

### What Is the Regulatory Intensity Impacting Private Forest Landowners in Each State?

**Fig. 1:**
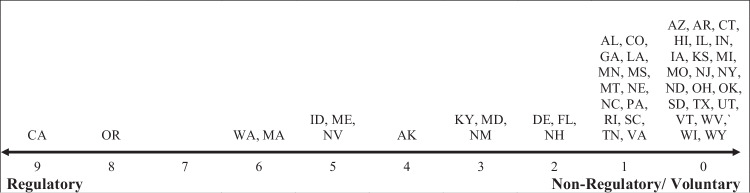
States by regulatory intensity, or the number of regulated forest practices on private forestland in the United States

To identify the intensity of practices regulated across the United States, we tabulated the number of regulations directly impacting forest practices in private forestland in every state. Results ranged from 0 to 9 and highlight the diversity of state approaches to ensuring sustainable forest management, based on our definitions and assessment as detailed in Table 2. In general, western states such as California (9 out of 12), Oregon (8 out of 12), and Idaho (5 out of 12) regulated more of the forest practices considered than states in the Southeast or East, except for Maine and Massachusetts (Fig. 1). Many states across the country regulate 2 or 3 forest practices (e.g., Alabama, Maryland, and Connecticut) while many states in the central part of the country do not uniformly regulate any forest practices at all. Instead, these states primarily rely on voluntary landowner compliance with BMPs or use incentive programs to encourage sustainable forest management (Kelly and Crandall 2022). It is important to remember that we used a strict definition of regulation: only those regulations that restrict the decision-making of landowners in all circumstances, as described in the introduction and in the specific guiding question column of Table 2, were given a value of 1. We recognize that other interpretations of the regulatory intensity for a state may differ. Also, we did not encounter policies that applied differently to small vs. large (or non-corporate vs. corporate) landowners. However, it is worth noting that the very recent changes in Oregon’s FPA that occurred after data collection for this paper offer some differences in riparian buffers that can apply to small vs. large landowners. These emerging aspects highlight the evolving nature of this regulatory landscape. A full tally of practices regulated by states can be found in Supplementary Table 1.

### Is There a Relationship Between State Socioeconomic or Political Characteristics and the Regulatory Intensity (i.e., Number) of Private Forest Practices?

Table 4 displays the variation in data used for the regression of state characteristics on regulatory intensity for United States as a whole and for different regions^1^: Northeast, Midwest, South, and West. These states within the regions are similar in terms of historical development, population characteristics, economic and political aspects and have been used as standardized data tabulation units in census bureau tables and publications.

**Table 4:** Summary statistics of the variables used across regions

Variables	US	Northeast	Midwest	South	West
Number of regulated forest practices (intensity, dependent variable)	0–9 (1.54)	0–6 (1.7)	0–1 (0.2)	0–3 (1.1)	0–9 (3.2)
Percent private land of all forestlands	3.6–93.9 (64.8)	47.9–90.7 (71.5)	40.4–92.8 (74.4)	64.6–93.8 (84.2)	3.6–42.7 (27.5)
Percent corporate forestland	0–90.6 (27.4)	18.1–62.7 (26.0)	0–23.0 (9.2)	17.4–63.2 (32.3)	4.5–90.6 (38.9)
Percent GDP	0.2–5.2 (1.76)	0.6–5.2 (1.7)	0.7–5.1 (1.7)	0.4–5 (2.3)	0.2–3.7 (1.2)
Environmental voting records of the members of Congress	0–100 (38.6)	36–100 (75)	0–57 (26.5)	1–92 (27.2)	0–100 (38.5)
Initiated statutes^a^	0–1 (0.42)	0–1 (0.2)	0–1 (0.5)	0–1 (0.1)	0–1 (0.8)

Values shown are min–max with (mean) in parentheses

^a^Mean for initiated statutes is proportion of states that are 1 in the certain region

Overall, there were few states that regulated multiple forest practices; most states relied on more voluntary approaches (mean intensity or cumulative number of regulations = 1.5). The states with higher regulatory intensity were concentrated in the West (maximum regulatory intensity 9 with mean of 3.2) and some in the Northeast (mean intensity of 1.7). The remaining two regions (Midwest and South) mostly opted for voluntary approaches (mean intensity 0.2 and 1.1, respectively). As anticipated, the percentage of private forestland and corporate ownership was highly variable between regions. The West had the lowest proportion of forestland in private hands (mean of 27.5%), including low proportions in states like Nevada (4%), while the South had the highest (mean = 84.2%). The proportion of private forestland in corporate ownership ranged from a high of 91% in Alaska (including tribal corporations) and Hawaii (66.4%) to essentially 0% in North Dakota. Corporate forestland ownership was concentrated in the western states (Alaska, Hawaii) and in a few eastern states (Maine, Florida); very little corporate forestland was found in Midwest states (North Dakota, Kansas).

Contribution of the forest products industry to overall gross state products ranged from 0.2 to 5% with little variation within the regions, except for a relatively lower contribution in the West. The forest product industries of Maine, Wisconsin, Arkansas, and Mississippi contribute the highest proportion to their state economies and all of these states fall in regions outside the West.

While in some states the members of the House of Representatives vote uniformly in support of environmental issues such as clean air and water, energy, climate change, environmental justice, public health, and other environmental programs, some state legislators average no support at all (the voting record agreement with the LCV ranged from 0 to 100). Most of the states with high values are concentrated in the Northeast (mean = 75) and the West (mean = 38.5). Our measure of direct democracy was a binary variable; the mean in Table 4 represents the proportion of states with direct democracy.

Results of the regression between regulatory intensity of forest practices on private forestland and state characteristics are shown in Table 5. Given the quasi-Poisson distribution and the regression model used, the estimates and standard errors are reported; those that are significant at a 5% significance level are indicated with an asterisk and the sign shows the direction of the relationship.

**Table 5:** Relationship between regulations in forest practices on private forestland and state characteristics

Variables	Estimate	Std. error
Intercept	−0.94	0.64
Percent private forestland (−)	−0.011	0.008
Percent corporate forestland (+)	0.019*	0.007
Percent GDP (+)	0.096	0.11
Environmental voting records of the member of Congress (+)	0.016*	0.005
Initiated statutes (+)	0.843*	0.372

^*^p ≤ 0.05

The overall percentage of private forestland was statistically insignificant although negatively related to the number of forest practices. On the other hand, percentage of corporate forestland (out of private forestland) was significant and positively related to the number of regulations of forest practices. The environmental voting records of the members of Congress, as well as our measure of direct democracy, were statistically significant and positively related to the number of forest practice regulations. Though GDP was statistically insignificant, it was positively related to the regulations of forest practices.

## Discussion

Our findings suggest that political and socioeconomic variables within states, including landownership patterns and political affiliation of elected representatives, impact private forestry regulations. Both the extent and intensity of policy tools used to achieve desirable outcomes from private forestland vary widely, and there is no one “right way” to get sustainable outcomes, even in states that share an economic reliance on the industry. Our regulatory intensity variable, with a mean of 1.54 out of 9, highlights that most states strongly support voluntary approaches that empower landowners (e.g., assistance programs) over regulatory approaches (Kreye et al. 2019). This finding supports previous studies indicating that private land policy in the United States has been mostly non-regulatory (Cubbage 1991; Schaaf and Broussard 2006; Janota and Broussard 2008). In the following paragraphs, we discuss the relationship of the independent variables with regulatory intensity, examining their implications in the context of previous literature.

Results showed a negative association between proportion of private (vs. public) forestland ownership and intensity of forest practices regulation, though statistically insignificant. The negative relationship is consistent with our expectations as states with low numbers of regulations are concentrated in regions with high levels of private ownership (e.g., the Southeast). The lack of significance may reflect that private forests consist of two very different ownership types, corporate, and non-corporate, and that combining these two types impacts significance.

Indeed, we found a significant, positive relationship between proportion of private corporate forestland and the intensity of forest practices regulations. This result supports our hypothesis, although it is unclear if the outcome is related to mistrust of corporate ownership behaviors and motivations, or if it is a logical response to more intensive forest management (and thus more negative externalities) practiced by corporate landowners compared to non-corporate landowners. Conversely, this result could be related to Cubbage and Newman’s (2006) finding that corporations are more actively pursuing proactive environmental agendas, possibly to mitigate negative public perception; for example, Oregon recently witnessed the development of expanded forest practice rules (the Oregon Private Forest Accord) proposed jointly by environmental groups and corporate landowners (ODF 2022). Regardless, states with private forestland dominated by corporate owners (much of the West and Maine) regulate more aspects of forest practices than regions dominated by non-corporate ownership.

There is also the consideration of cost to landowners, as regulations to ensure socially desirable outcomes from forest management may come at a loss in terms of revenue maximization. The use of regulations encompasses a tradeoff between economic efficiency and environmental outcomes. The impacts of this tradeoff are different between small non-corporate and large corporate landowners. It may be easier to regulate behavior of large corporate landowners who have more resources; non-corporate landowners, who typically own fewer acres, may not be able to absorb the costs of regulation. In addition, regulations are more likely to push small landowners out of forest management altogether (Mehmood and Zhang 2001), an outcome not in states’ interest; thus, states with high levels of non-corporate landowners may moderate the role of regulatory instruments. Finally, small landowners may be presumed to manage based on intrinsic land ethics, justifying less regulatory approaches (Kelly and Crandall 2022).

Contrary to our hypothesis, no significant difference was found between the number of regulated forest practices and the economic importance of the forestry industry (defined as share of GDP). This lack of statistical signal may be due to the fact that states among the top in terms of forest GDP have very different regulatory practices, pulling the variable in both directions. For example, Maine and Oregon are highly regulatory whereas Wisconsin and Arkansas are non-regulatory in nature, despite all having substantial GDP from forest industry. In other ways as well, the incentives linking regulations to economic dependence may be more complex. A state with a high GDP from forest industry might have pressure to both rely on hands-off (non-regulatory) programs to encourage continued investment in the forest sector, or pressure to regulate more due to the higher environmental impacts related to management activity. This relationship between economic dependence and pressures for more or less regulation may play out differently in different states; for example, the environmental pressure related to a given harvest intensity is not the same in areas of different harvest cost, forest species, topography, and climate. It would be interesting for future studies to elaborate on this tension between incentives for high and continued investment and returns vs. incentives to minimize negative externalities and land management impacts on a state-by-state basis, where the competing effects are not canceled out.

The association between direct democracy and the number of regulated forest practices was statistically significant and positive. All of the states regulating higher number of forest practices (e.g., California, Oregon, Washington, Idaho, and Maine) have provision of direct democracy. Numerous examples can be drawn that reflect this relationship. In Maine, public support for ballot measures that would ban clearcutting in 1996 incentivized the state Governor’s office and legislature to propose an alternate referendum, representing a compromise solution.

The environmental voting record of members of the House of Representatives was positive (as anticipated) and statistically significant. In states with more environmentally minded elected representatives, there is a correspondingly high use of multiple regulations on forest practices. This relationship holds across environmentally minded states with high proportions of corporate forestland (e.g., Oregon) as well as states with low proportions of corporate forestland (e.g., Massachusetts). In this way, forestry regulations appear to be aligned with other pro- or anti-environmental voting records; the higher the score on environment friendliness of the state’s legislators, the more sympathetic they are to environmental causes, increasing the likelihood of a high intensity of regulations favoring public resources in those states (Mehmood and Zhang 2001). In contrast, lower voting agreement with the LCV might indicate states where legislators favor protection of private property rights of private forest landowners.

Finally, in the process of collecting data we observed that states regulate *different* forest practices – a fact that was not evident in the intensity index we created. For example, while Kentucky and Maryland each regulate 3 of the 12 forest practices, Kentucky requires a burn manager at the side while conducting prescribed burning whereas Maryland requires notification to harvest trees; the scope of these thus may not impact the operating environment of landowners equally between states. In addition, though Kentucky is not a very regulatory intensive state, the Kentucky Forest Conservation Act (Kentucky Revised Statutes, KRS 149.330 to 149.355 of 1998) *requires* the use of Kentucky’s BMPs including presence of a master logging on every commercial logging operation (KRS 149.330 to 149.355 1998). Rather than a linear axis of regulatory intensity from low to high, the experience of private landowners in navigating forest regulations is likely more nuanced and multidimensional, encompassing the number of regulated forest practices, the types of forest practices regulated, and which agency regulates each forest practice. Future studies may explore this more complex relationship between regulation and private forest management.

## Conclusion

While this study has conducted one of the first assessments to our knowledge of the regulatory intensity of states, there are many opportunities for future research to confirm these results with updated data or to explore other facets of these regulations, and we acknowledge there are many approaches to unpack regulations concerning forest practices. For example, although both Washington and California have clearcut size limits and so received a score of 1 for regulating harvesting, Washington limits clearcut sizes to 120 acres while California to 40 acres (with some lower limits depending on harvest type). Articulating these differences between states would require a deeper dive into the specifics of forest regulations, an area of fruitful future research. In another example, Sun and Tolver (2012) focused on 28 specific questions regarding prescribed burning alone, within a limited number of states. In contrasts, our analysis of prescribed burning is limited to evaluating presence of personnel and/or requirement of a burn permit. Our categories of regulations on forest practices were intentionally narrow and specific. For this study we chose to forego more varied delineation of types of regulations per forest practice in order to capture all 50 states. In addition, the use of observed data available at the national level limits us from understanding the nature of differing levels of regulation on actual private landowner behavior. Future studies comparing the different experiences of landowners by regulatory environment – comparing California to Texas, for example – would help us understand if all systems are allowing sufficient landowner flexibility to ensure that sustainable forest management continues to be practiced.

Regulations are always changing, and this study provides an overview of observed state-level forest regulation and socioeconomic and political context at a moment in time. We cannot deduce any causality in these relationships; for example, does increasing environmental mindedness result in more regulations, or does a more regulatory environment create more willingness for further regulation, perhaps because of the demonstrated effectiveness of those regulations? To untangle this relationship would require establishing timelines of both changes in forest policy and voting records (or other variables of interest), something that may be suited to future case-study approaches. Some states make frequent changes to regulations, and some do not change for decades.

It is important to highlight that we were limited to five state-level socioeconomic and political variables when characterizing the influence of these factors on regulation of forest practices across the United States. There is an opportunity for future researchers to explore additional aspects of individual state characteristics with regulatory intensity. Future researchers could explore other variables or use different aspects of our (rather broadly defined) variables. For example, to gauge political context with more nuance, researchers could use variables such as number of environmental interest groups in the state, state agency capacity both in terms of budgeting and human resources, and state-level legislative political environment (e.g., proportion of Republicans and Democrats in the state legislature). Researchers in other countries would by necessity use socioeconomic and political variables relevant to their contexts.

Another avenue for future research is the role of regulation in mitigating negative externalities that arise from private forest management actions. The data we used here do not allow us to understand whether or not the actual level of externalities varies by state, and thus if the state-level variations in policy are logical outcomes of environmental impacts. For example, it could be that environmental impacts from management are higher in states that ultimately end up with more intense levels of regulation (e.g., California and Oregon as compared to Georgia and Texas).

By establishing a baseline count of regulated practices across all 50 states and analyzing state factors related to that intensity, this study starts to dissect and help us explain *why* policies differ, not just how they differ. These findings will be useful to compare across different jurisdictions, where other socioeconomic and political variables may logically be related to regulatory intensity. Our study provides evidence about how forestry policies are impacted by the broader socioeconomic and political contexts in which they are formulated, and this is important for private landowners and the goods and services provided by their forestlands.

### Supplementary Information

Supplementary Information

## Footnotes

1 Northeast = Connecticut, Maine, Massachusetts, New Hampshire, Rhode Island, Vermont, New Jersey, New York, and Pennsylvania; Midwest = Indiana, Illinois, Michigan, Ohio, Wisconsin, Iowa, Kansas, Minnesota, Missouri, Nebraska, North Dakota, South Dakota; Southeast = Delaware, District of Columbia, Florida, Georgia, Maryland, North Carolina, South Carolina, Virginia, West Virginia, Alabama, Kentucky, Mississippi, Tennessee, Arkansas, Louisiana, Oklahoma, Texas; West = Arizona, Colorado, Idaho, New Mexico, Montana, Utah, Nevada, Wyoming, Alaska, California, Hawaii, Oregon, and Washington.
