# Supplementary material for: Regulatory Intensity on Private Forestland and its Relationship with State Characteristics in the United States
Source: Environ Manage. 2024 May 6;73(6):1121–33. doi: 10.1007/s00267-024-01974-6 (PMC11136758; doi:10.1007/s00267-024-01974-6)
Supplement: Supplementary file 1 — Supplementary Information [file 267_2024_1974_MOESM1_ESM.docx]

**Supplementary Table 1. States with regulations (as defined by the authors) for each forest practice category.**

|  | **Water Quality** | **Reforestation** | **Timber Harvesting** | **Management and Planning** | **Notification and Permit** | **Infrastructure** | **Wildlife and Endangered species** | **Prescribed Burning** | **Carbon** | **Aesthetic** | **Total** |
| --- | --- | --- | --- | --- | --- | --- | --- | --- | --- | --- | --- |
| **Alabama** |  |  |  |  |  |  |  | 1 |  |  | 1 |
| **Alaska** | 1 | 1 |  | 1 |  |  |  | 1 |  |  | 4 |
| **Arizona** |  |  |  |  |  |  |  |  |  |  | 0 |
| **Arkansas** |  |  |  |  |  |  |  |  |  |  | 0 |
| **California** | 1 | 1 | 1 | 1 | 1 | 1 | 1 | 1 | 1 |  | 9 |
| **Colorado** |  |  |  |  |  |  |  | 1 |  |  | 1 |
| **Connecticut** |  |  |  |  |  |  |  |  |  |  | 0 |
| **Delaware** | 1 |  |  |  | 1 |  |  |  |  |  | 2 |
| **Florida** | 1 |  |  |  |  |  |  | 1 |  |  | 2 |
| **Georgia** |  |  |  |  |  |  |  | 1 |  |  | 1 |
| **Hawaii** |  |  |  |  |  |  |  |  |  |  | 0 |
| **Idaho** | 1 | 1 |  |  | 1 | 1 |  | 1 |  |  | 5 |
| **Illinois** |  |  |  |  |  |  |  |  |  |  | 0 |
| **Indiana** |  |  |  |  |  |  |  |  |  |  | 0 |
| **Iowa** |  |  |  |  |  |  |  |  |  |  | 0 |
| **Kansas** |  |  |  |  |  |  |  |  |  |  | 0 |
| **Kentucky** | 1 |  |  |  |  | 1 |  | 1 |  |  | 3 |
| **Louisiana** |  |  |  |  | 1 |  |  |  |  |  | 1 |
| **Maine** | 1 | 1 | 1 |  | 1 |  |  | 1 |  |  | 5 |
| **Maryland** | 1 |  |  |  | 1 |  |  | 1 |  |  | 3 |
| **Massachusetts** | 1 | 1 | 1 |  | 1 | 1 |  | 1 |  |  | 6 |
| **Michigan** |  |  |  |  |  |  |  |  |  |  | 0 |
| **Minnesota** |  |  |  |  |  |  |  | 1 |  |  | 1 |
| **Mississippi** |  |  |  |  |  |  |  | 1 |  |  | 1 |
| **Missouri** |  |  |  |  |  |  |  |  |  |  | 0 |
| **Montana** | 1 |  |  |  |  |  |  |  |  |  | 1 |
| **Nebraska** |  |  |  |  |  |  |  | 1 |  |  | 1 |
| **Nevada** | 1 | 1 |  |  | 1 |  | 1 | 1 |  |  | 5 |
| **New Hampshire** | 1 |  |  |  |  |  |  | 1 |  |  | 2 |
| **New Jersey** |  |  |  |  |  |  |  |  |  |  | 0 |
| **New Mexico** | 1 | 1 |  |  | 1 |  |  |  |  |  | 3 |
| **New York** |  |  |  |  |  |  |  |  |  |  | 0 |
| **North Carolina** |  |  |  |  |  |  |  | 1 |  |  | 1 |
| **North Dakota** |  |  |  |  |  |  |  |  |  |  | 0 |
| **Ohio** |  |  |  |  |  |  |  |  |  |  | 0 |
| **Oklahoma** |  |  |  |  |  |  |  |  |  |  | 0 |
| **Oregon** | 1 | 1 | 1 |  | 1 | 1 | 1 | 1 |  | 1 | 8 |
| **Pennsylvania** |  |  |  |  |  |  |  | 1 |  |  | 1 |
| **Rhode Island** |  |  |  |  | 1 |  |  |  |  |  | 1 |
| **South Carolina** |  |  |  |  |  |  |  | 1 |  |  | 1 |
| **South Dakota** |  |  |  |  |  |  |  |  |  |  | 0 |
| **Tennessee** |  |  |  |  |  |  |  | 1 |  |  | 1 |
| **Texas** |  |  |  |  |  |  |  |  |  |  | 0 |
| **Utah** |  |  |  |  |  |  |  |  |  |  | 0 |
| **Vermont** |  |  |  |  |  |  |  |  |  |  | 0 |
| **Virginia** |  |  |  |  | 1 |  |  |  |  |  | 1 |
| **Washington** | 1 | 1 | 1 |  | 1 | 1 |  | 1 |  |  | 6 |
| **West Virginia** |  |  |  |  |  |  |  |  |  |  | 0 |
| **Wisconsin** |  |  |  |  |  |  |  |  |  |  | 0 |
| **Wyoming** |  |  |  |  |  |  |  |  |  |  | 0 |
